# Peri-hand space representation in the absence of a hand – Evidence from congenital one-handers

**DOI:** 10.1016/j.cortex.2017.08.016

**Published:** 2017-10

**Authors:** Roni O. Maimon-Mor, Heidi Johansen-Berg, Tamar R. Makin

**Affiliations:** a FMRIB Centre, Nuffield Department of Clinical Neuroscience, University of Oxford, Oxford OX39DU, United Kingdom; b Institute of Cognitive Neuroscience, University College London, London WC1N 3AZ, United Kingdom

**Keywords:** Peripersonal space, Body representation, Amputees, Neuroimaging, Multisensory integration, Spatial representation

The space immediately surrounding us is known to be represented relative to our hands, i.e. in a hand-centred coordinate system (peri-hand space, PHS) (Graziano, Hu, & Gross, 1997; for review see; Brozzoli, Makin, Cardinali, Holmes, & Farnè, 2012). Individuals born without a hand (hereafter one-handers) manually interact with the world differently, potentially altering the representation of their near space. Previous behavioural research on PHS representation in one-handers and acquired amputees found evidence of a mild visuospatial bias against their missing hand side (Makin, Wilf, Schwartz, & Zohary, 2010), suggesting that hand loss impacts visual processing.

Here, we examined the representation of peri-(missing)-hand space (P[M]HS) in one-handers, by interrogating their PHS network using functional neuroimaging. The PHS network is comprised of several brain regions, most prominently: the anterior and posterior parts of the intraparietal sulcus (IPS), the lateral occipital cortex (LOC), the supramarginal gyrus and the pre-motor cortex (Brozzoli et al., 2013, Brozzoli et al., 2011, Makin et al., 2007). A subset of this network is sensitive to visual features of the hand position, rather than its veridical position (e.g., LOC and posterior IPS) (Makin et al., 2007).

If the PHS network is specifically anchored to the hand, then the absence of a hand should result in diminished P[M]HS representation (and possibly enhanced representation in relation to the intact hand). Moreover, areas known to selectively respond to visual features of the hand (e.g., posterior IPS) should not be activated when objects are approaching the missing hand. If, however, PHS network is defined by the zone for object manipulation (Brozzoli et al., 2014, Makin et al., 2012), representation should be tied to the participant's ability to interact with objects, and anchored to whichever actuator supplements the missing hand function. Under this framework, diminished P[M]HS representation would result from the reduced ability to interact with objects.

**Fig. 1:**
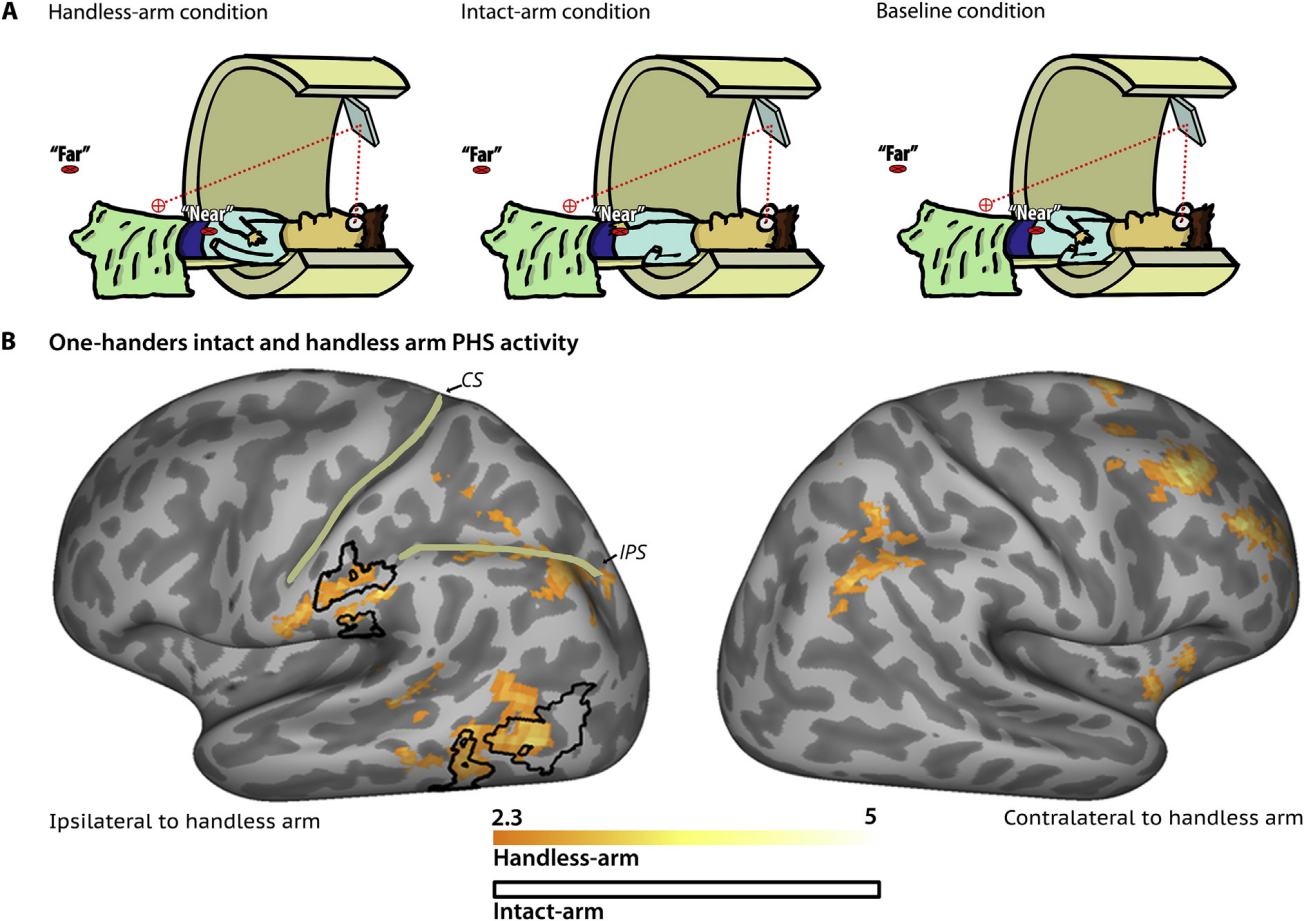
(A) A schematic illustration of the experimental conditions. In each condition, a moving object was presented in one of two positions, near or far. The participant maintained fixation between the positions throughout the experiment (Fixation point indicated by red cross-hairs; line of sight, via a mirror, indicted by dotted red line). Arm positions changed between conditions as follows: Baseline condition – both arms were retracted, Handless-arm condition – intact-arm retracted and handless-arm extended, Intact-arm condition – intact-arm extended and handless arm retracted. (B) Visual selectivity for the space surrounding the handless or intact arms in one-handers. The hemisphere contralateral to the intact hand is presented on the left and the hemisphere contralateral to the missing hand on the right. Areas showing greater differential near > far activity in handless-arm condition over baseline were found in both hemispheres and are presented in an orange-yellow gradient (z values are indicated in the bottom). Areas showing greater differential near > far activity in intact-arm condition over baseline were found in the hemisphere contralateral to the intact hand only, and are presented in black lines. Clusters are defined using family-wise-error-corrected cluster significance threshold of *p* < .05. Unthresholded Z-maps can be found online at: https://neurovault.org/collections/2119/.

We examined PHS representation in 10 one-handers born with one hand due to congenital upper-limb below-elbow deficiency; Table S1 in the Supplementary Material. We further compared PMHS with an additional group of below-elbow unilateral amputees (*n* = 9) for control purposes. Within each functional run a 3D moving object appeared in one of two locations: either near or far from the participants' body, as used in previous studies (Brozzoli et al., 2011, Brozzoli et al., 2013, Makin et al., 2007). In order to evaluate the PHS network of a single hand the spatial relationship between the visual stimuli and participants' upper limbs differed across runs: (1) handless-arm visibly extended towards the near stimulus, intact hand retracted and (2) intact-arm visibly extended towards the near stimulus, handless-arm retracted (Fig. 1A, see Supplementary Methods for further details). In addition, a separate baseline run was acquired in which both arms were retracted away from the visual stimuli. Each of these three runs was repeated twice. To identify brain areas that show greater activity to objects positioned close to the hand a near > far contrast was calculated within each of the arm runs. To control for differences in visual features between near and far positions that are not PHS relevant, this was contrasted with the near > far contrast of the baseline runs. Therefore, PHS network was identified for each arm using the contrast [arm (near > far)] > [baseline (near > far)].

We found strong and widespread activity in the PHS network anchored to the handless-arm of one-handers (P[M]HS; Fig. 1B). In particular, differential activity was identified in PHS regions such as anterior IPS and supramarginal gyrus ipsilaterally to the handless arm, but also in the posterior IPS (bilaterally) and LOC (ipsilaterally), thought to be sensitive to visual features of hand position (Fig. 1B, Table S2). The P[M]HS activity profile overlapped with PHS network activity for the intact hand (Black contour in Fig. 1B, Table S3) and was consistent with previous reports of PHS in individuals with two hands (Brozzoli et al., 2011, Makin et al., 2007). This suggests that spatial representation in those areas is anchored to the position of the handless-arm, irrespective of the actual physical absence of the hand itself.

We next determined how one-handers' P[M]HS representation compares with another population with a hand-loss. We therefore compared P[M]HS in one-handers and in acquired amputees undergoing the same experimental procedure (Figure S1). While one-handers and amputees both have one-hand, they show important brain and behavioural differences (Makin et al., 2013) relevant for P[M]HS representation (see Supplementary methods and discussion). For example, as demonstrated by the current study cohort, acquired amputees show reduced functionality of their handless-arm in daily tasks compared to one-handers (Mann–Whitney *U* = 8.5, *p* = .002). A group comparison for near > far contrast in the handless-arm condition revealed increased activity in a subset of PHS network in one handers, relative to acquired amputees (see Supplementary figure S2). This suggests that P[M]HS representation does not automatically remap to the handless arm.

Our main finding shows that the P[M]HS evoked activity is present in one-handers despite congenital hand-loss. We suggest that PHS network, normally representing the space surrounding the hand, can adapt to represent the space surrounding the handless-arm in the absence of a hand. This reanchoring of PHS in one handers, and the finding of greater P[M]HS activity in one handers compared to amputees, can be interpreted differently under two existing frameworks. First, considering the evidence for PHS as an interface for effector-centred representation, and the suggested remapping of PHS during prosthesis (Canzoneri, Marzolla, Amoresano, Verni, & Serino, 2013) and tool use (Martel, Cardinali, Roy, & Farnè, 2016) the increased use of the handless arm in one-handers should lead to a greater representation of P[M]HS in one-handers compared to acquired amputees. This interpretation is consistent with recent findings showing that high daily usage of the residual arm in one handers associates with increased arm representation in the sensorimotor system (Hahamy et al., 2015, Hahamy et al., 2017). Alternatively, the reanchoring of P[M]HS representation to the arm (instead of the hand) might only be possible during the developmental period of the visual system (Wandell & Smirnakis, 2010), leading to greater P[M]HS activity for congenital one-handers than for acquired amputees. This interpretation introduces new considerations for the ability of PHS representation to dynamically change. Since one-handers use their handless-arm to interact with objects and experienced handlessness from early development, it is difficult to determine which framework provides better interpretation to our results.
