# Supplementary material for: Peri-hand space representation in the absence of a hand – Evidence from congenital one-handers
Source: Cortex. 2017 Oct;95:169–71. doi: 10.1016/j.cortex.2017.08.016 (PMC5637313; doi:10.1016/j.cortex.2017.08.016)
Supplement: Supplementary file 3 [file mmc3.pdf]

Supplementary Material

Methods

Participants

Ten one-handers with congenital upper-limb below-elbow deficiency (mean age±s.e.m = 33.8±4, seven missing their left hand, six females) and nine acquired one-handed below-elbow amputees (age = 42±4, six missing their left hand, two females) were recruited to take part in a study through the Oxford Centre for Enablement and Opcare in accordance with NHS National Research Ethics Service Approval (10/H0707/29), and written informed consent was obtained. The participants took part in a large-scale study, involving multiple tasks and further participants, as previously reported (Hahamy et al., 2015; Makin, Cramer, et al., 2013; Makin, Filippini, et al., 2015; Makin, Scholz, et al., 2013; Makin, Scholz, Henderson Slater, Johansen-Berg, & Tracey, 2015). The subset of participants included in our analysis were chosen based on the level of their limb deficiency (below the elbow level of amputation), to reduce inter-subject and inter-group variability. No significant age differences were found between groups ($t(1,17) = 1.421$, $p = 0.173$). Data from one additional acquired amputee was discarded from analysis, owing to excessive head movements (see Data analysis for more details). Below we detail the experimental procedures directly relating to the reported results.

Handless-arm related measurements

Use of the handless-arm in one-handers was assessed with a revised version of the Motor Activity Log (Uswatte, Taub, Morris, Light, & Thompson, 2006) as described before (Makin, Cramer, et al., 2013). In short, participants were requested to rate how frequently they incorporate their handless-arm (either directly, or by using a prosthesis) in an inventory of daily activities, with varying degrees of motor control. This questionnaire, indexing bimanual usage, was previously validated using limb acceleration data (Makin, Cramer, et al., 2013) and behavioural lab testing (Hahamy et al., 2017), collected in ecological settings. Participants also rated their prosthesis wearing frequency on a scale: 0-never, 1-rarely, 2-occasionally, 3-daily (<4 hours), 4-daily (4-8 hours), 5-daily (>8 hours). (Table S1). Some participants use more than one type of prosthesis, in that case the data from the most frequently used prosthesis is shown. In addition, vividness/intensity of phantom sensations and pain ratings were collected, as previously described (Makin, Scholz, et al., 2013, 2015)

Task and Stimuli

Participants lay in the magnetic resonance imaging (MRI) scanner and viewed the apparatus through a mirror placed above their faces. Two targets (made of cardboard) were presented in the participant's field of view: one was set on the participant's torso, where she could comfortably place her intact hand ("near" target). The second target ("far" target) was set 1 meter away from the near target towards the feet. (This paradigm has been shown to be successful in targeting the peri-personal space network (Makin, Holmes, & Zohary, 2007). The visual stimulus was a ball attached to a 70-cm-long stick, which was moved toward (and stopping 2–5cm from the target) and away from one of the targets at a frequency of ~1 Hz. The visual stimuli were delivered by a trained experimenter, who listened to a metronome with a frequency of 1 Hz. The participant could not see the experimenter's hand and could see only the moving ball attached to the stick. During the stimulation periods, participants were required to determine whether the trajectory of the ball would hit the center of the target or not, by covertly responding "yes" or "no". Eye movements were not monitored during scanning.

This procedure was repeated under 3 experimental settings (Fig. 1 in main text). In the "baseline condition", both intact and handless arms were retracted toward the shoulders (Fig. 1A). In the "handless-arm condition" the participant's handless-arm (residual limb) was extended towards the near target, since these participants had a relatively low amputation the handless-arm was visible to the participant, the intact-arm was retracted in the same position as in the baseline condition (Fig. 1B). In the "intact-arm condition," the participant's intact-arm and hand was extended, the near target was positioned on the participant's hand, the handless-arm was retracted in the same position as in the baseline condition (Fig. 1C).

Experimental design

Conditions were changed between functional runs and were presented in the following order (regardless of the missing hand side): left arm extended, baseline (both arm retracted), right arm extended. This order was repeated twice resulting in two runs for each condition (we note that for one congenital one-hander only a single handless-arm functional run was acquired, due to time constraints. Within each run two types of trials (ball approaching near or far targets) were presented in pseudo-random order and repeated seven times per run. Each trial lasted 12 seconds and was followed by a rest period of 8 seconds. The experiment began and ended with a rest period of 14 seconds. The temporal order of trial types (near and far) was kept constant across conditions.

MRI data acquisition

The MRI measurements were obtained using a 3-Tesla Verio scanner (Siemens, Erlangen, Germany). Anatomical data were acquired with a 32-channel head coil using a T1-weighted magnetization prepared rapid acquisition gradient echo sequence with the parameters: TR=2040 ms; TE=4.7 ms; flip angle=8° and voxel size=1 mm isotropic resolution. Functional data based on the blood oxygenation level-dependent signal were acquired with a 12-channel coil (allowing a better view of the mirror reflecting the body) using a multiple gradient echo-planar T2*-weighted pulse sequence, with the parameters: TR=2000 ms, TE=30 ms, flip angle=90°, imaging matrix=64x64 and FOV=192 mm axial slices. Thirty-six slices with slice thickness of 3mm and no gap were oriented in the oblique axial plane, covering the whole cortex, with partial coverage of the cerebellum.

Data analysis

Functional MRI data processing was carried out using FEAT (FMRI Expert Analysis Tool) Version 6.00, part of FSL (FMRIB's Software Library, www.fmrib.ox.ac.uk/fsl). Data collected for individuals with absent right hands (2 acquired and 3 congenital participants), were mirror reversed across the mid-sagittal plane prior to all analyses so that the hemisphere corresponding to the missing hand was consistently aligned. This was performed since some key elements of the network may be lateralised to the hand (Cooke & Graziano, 2003; Makin et al., 2007). This procedure was further validated post-hoc, as described below. Registration of the functional data to the high resolution structural image was carried using the boundary based registration algorithm (Greve & Fischl, 2009). Registration of the high resolution structural to standard space images was carried out using FLIRT (Jenkinson, Bannister, Brady, & Smith, 2002; Jenkinson & Smith, 2001) and was then further refined using FNIRT nonlinear registration (Andersson, Jenkinson, & Smith, 2007a, 2007b). The following pre-statistics processing was applied; motion correction using MCFLIRT (Jenkinson et al., 2002); non-brain removal using BET (Smith, 2002); spatial smoothing using a Gaussian kernel of FWHM 5mm; grand-mean intensity normalisation of the entire 4D dataset by a single multiplicative factor; highpass temporal filtering (Gaussian-weighted least-squares straight

line fitting, with sigma=20s).Time-series statistical analysis was carried out using FILM with local autocorrelation correction (Woolrich, Ripley, Brady, & Smith, 2001). The time series model included near and far stimuli onsets convolved with a double gamma HRF function and their temporal derivatives. Six motion parameters were added as confound regressors. Indicator functions were added to model out single TR's identified to have excessive motion according to framewise displacement > 0.9. A separate regressor was used for each high motion TR.

A near > far contrast was calculated at the first level for each functional run. The second-level analysis which averaged contrast estimates over runs for each condition within subject was carried out using a fixed effects model. Group-level analysis was carried out using FLAME stage 1 and stage 2 (FMRIB's Local Analysis of Mixed Effects) (Beckmann, Jenkinson, & Smith, 2003; Woolrich, 2008; Woolrich, Behrens, Beckmann, Jenkinson, & Smith, 2004). A voxelwise Voxel Based Morphometry (VBM) regressor was added to the group level analysis to control for age and non-age related structural differences (The VBM data was taken from a previous analysis detailed in Makin, Scholz, et al., 2013). The volume morphometry measure was demeaned for each voxel across groups. Z (Gaussianised T/F) statistic images were thresholded using clusters determined by Z>2.3 and a (corrected) cluster significance threshold of P=0.05 (Worsley, 2001). For visualisation purposes, contrast group maps were projected onto inflated surface of an averaged brain using the surf_proj command (FSL). Figures were created using HCP Connectome Workbench visualization software (Marcus et al., 2011)

As mentioned above, MRI data acquired from three one-handers with a missing right hand were mirror reversed across the mid-sagittal plane to obtain group analysis that is lateralised to the missing hand. To test whether one-handers with absent right hands show a quantitatively different effect than one-handers missing their left hand (n=7), mean contrast activation values were extracted from areas showing a significant effect in one-handers in the P[M]HS contrast: [handless-arm (near>far)] > [baseline (near>far)]. If a right hand deficiency results in different activity pattern we would expect these participants to show relative lower values under the group mask. Ordering ascendingly one-handers with absent right hands are ranked 2nd,5th and 7th out of 10. A Mann Whitney test between the right and left one-handers result in U=8.00, p = 0.667. This analysis confirms that the flipping procedure did not falsely drive the results. Nevertheless, we note that the flipping procedure might have blurred other PMHS representation that is lateralised to the left/right hemisphere, rather than the effector.

ID	Gender	Cause of Amputation	Missing hand side	Handedness	Age	Years Since Amp.	Level of Amputation	Prosthesis usage	Prosthesis type	Residual arm use
A01	F	Trauma	L	R	43	4	Below elbow	Occasionally	Cosmetic	0.43
A02	M	Nerve Injury	R	L	42	19	Below elbow	Occasionally	Cosmetic	0.2
A03	F	Trauma	R	L	21	2	Below elbow	Never	Cosmetic	0.33
A04	M	Trauma	L	L	54	33	Wrist	Daily (>8 hours)	Cosmetic	0.37
A05	M	Infection	L	R	51	15	Below elbow	Daily (>8 hours)	Functional	0.3
A06	M	Trauma	L	R	22	3	Wrist	Never	Cosmetic	0.59
A07	M	Trauma	L	R	43	9	Below elbow	Daily (>8 hours)	Functional	0.44
A08	M	Trauma	L	R	50	21	Below elbow	Daily (>8 hours)	Cosmetic	0.35
A09	M	Trauma	L	R	52	6	Below elbow	Daily (>8 hours)	Functional	0.3
C01	F	Dysmelia	R	L	31	31	Below elbow	Daily (>8 hours)	Cosmetic	0.5
C02	F	Dysmelia	L	R	24	24	Below elbow	Daily (4-8 hours)	Cosmetic	0.63
C03	M	Dysmelia	L	R	35	35	Below elbow	Daily (>8 hours)	Cosmetic	0.63
C04	M	Dysmelia	L	R	31	31	Wrist	Never	Cosmetic	0.44
C05	F	Dysmelia	L	R	25	25	Below elbow	Never	Cosmetic	0.87
C06	M	Dysmelia	L	R	54	54	Below elbow	Daily (>8 hours)	Functional	0.74
C07	M	Dysmelia	L	R	49	49	Wrist	Never	Cosmetic	0.74
C08	F	Dysmelia	R	L	22	22	Below elbow	Rarely	Cosmetic	0.67
C09	F	Dysmelia	R	L	49	49	Below elbow	Daily (4-8 hours)	Cosmetic	0.39
C10	F	Dysmelia	L	R	18	18	Below elbow	Never	Cosmetic	0.39

Table S1. Demographic details of one-handed individuals with acquired (A) and congenital (C) hand loss. Handness = hand dominance prior to amputation (based on self-report), L = left, R = right; Years Since Amp. = Years since amputation; Prosthesis usage = Prosthetic limb usage frequency; Residual arm usage = Questionnaire ratings for residual arm usage in daily activities.

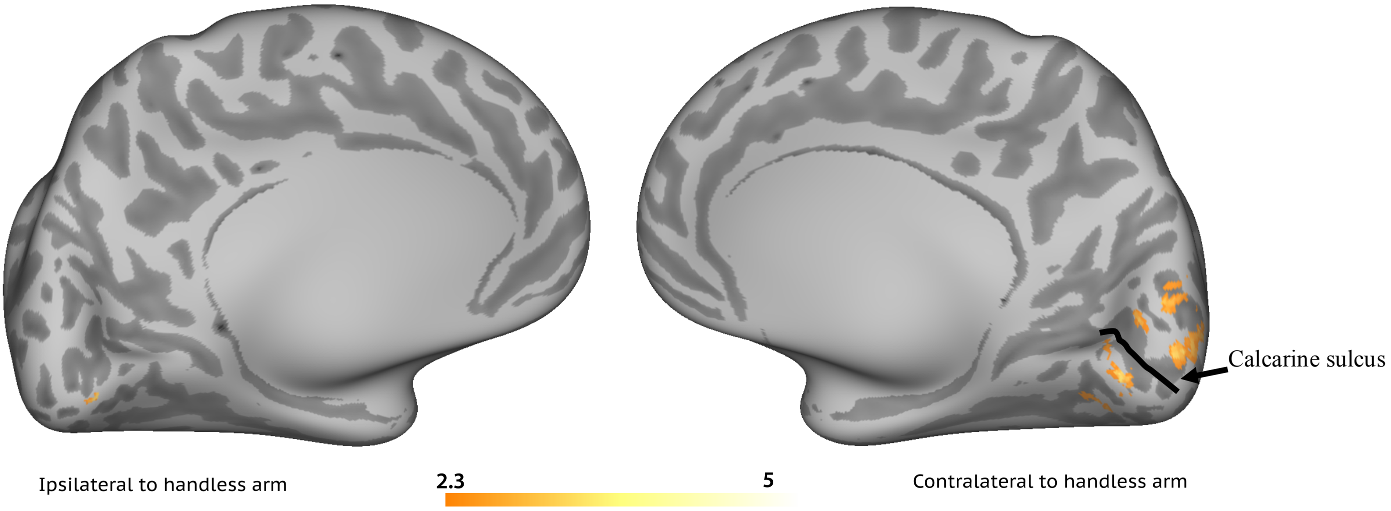

Figure S1. Visual selectivity for the space surrounding the handless arm of acquired amputees. The only areas showing greater differential near>far activity in handless-arm condition over baseline were identified in low-level visual cortex surrounding the calcarine sulcus (presented in an orange-yellow gradient, family-wise-error-corrected cluster significance threshold of $p<0.05$). No significant clusters were found on the lateral cortical surface.

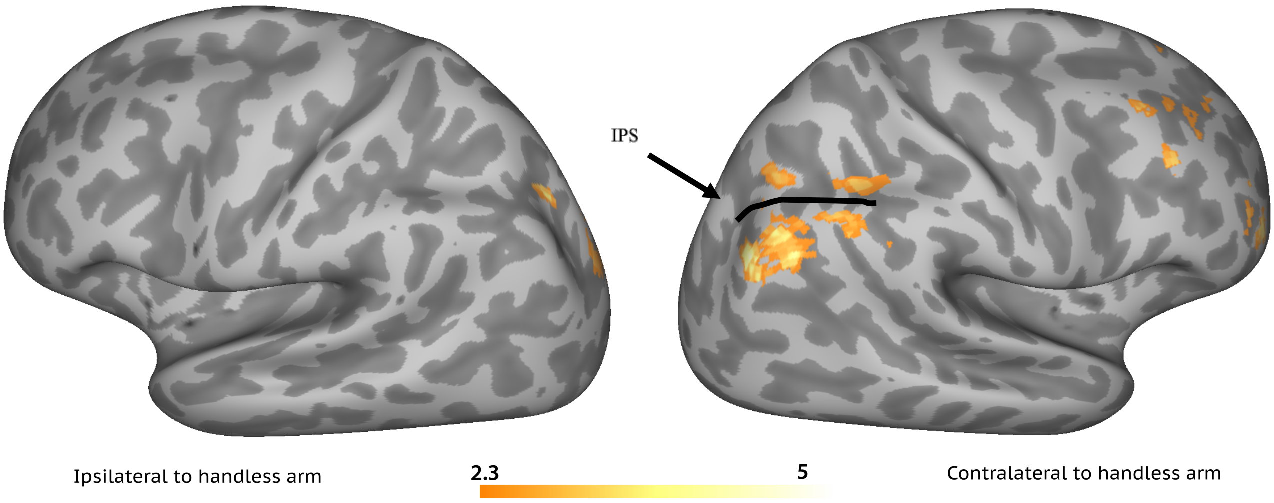

Figure S2. Greater visual selectivity for the space surrounding the handless arm in one-handers over acquired amputees. Areas showing greater differential near>far activity in handless-arm condition were identified along the intraparietal sulcus (IPS), as well as other areas, detailed in Table S4. (presented in an orange-yellow gradient; family-wise-error-corrected cluster significance threshold of $p<0.05$). Note that in the same contrast no areas showed greater differential activity in acquired amputees over one-handers.

Area	Z	MNI coordinates of local maxima		
		x	y	z
Right middle frontal gyrus	4.24	54	24	34
Right dorsolateral prefrontal	4.06	38	26	40
Right frontal pole	4.08	37	57	16
Left posterior superior temporal gyrus	3.78	-54	-44	12
Left lateral occipital cortex (V5)	3.3	-42	-70	4
Left inferior parietal cortex	3.58	-62	-32	38
Left inferior postcentral sulcus	3.2	-64	-12	22
Left Anterior IPS	3.81	-44	-48	56
Left transverse occipital/ posterior IPS	3.45	-28	-81	40
Bilateral posterior cingulate cortex	3.76	-10	-40	32
Right middle IPS	3.95	38	-50	50
Right anterior Insula	4.29	32	14	-10
Bilateral anterior cingulate cortex	3.6	0	6	32

Table S2: Contrast peak voxel coordinates within significant clusters for the P[M]HS contrast in one-handers: areas showing greater differential near>far activity in handless-arm condition over baseline

Area	Z	MNI coordinates of local maxima		
		x	y	z
Left superior lateral occipital cortex	3.97	-44	-80	18
Left lateral occipital cortex (v5)	3.8	-50	-76	3
Left anterior IPS	3.69	-56	-30	42

Table S3: Contrast peak voxel coordinates within significant clusters for the PHS contrast for the intact arm in one-handers: areas showing greater differential near>far activity in intact-arm condition over baseline

Area	Z	MNI coordinates of local maxima		
		x	y	z
Left superior occipital cortex	4.71	-18	-86	36
Right superior parietooccipital cortex	4.43	4	-32	71
Right anterior middle frontal gyrus	4.11	30	32	52
Right lateral occipital cortex	4.51	40	-74	38
Bilateral posterior Cingulate	3.53	-2	-38	38
Right Frontal Pole	3.63	36	44	12

Table S4. Group comparison peak contrast coordinates within significant clusters of voxels for greater differential near>far activity in one-handers over acquired amputees in the handless-arm condition.

ID	Chronic phantom pain magnitude	Phantom pain magnitude on scanning day	Chronic phantom sensations magnitude	Phantom sensations magnitude on scanning day	Telescoping
A01	7	0	10	10	Y
A02	2.5	0	1.74	5	N
A03	3.33	0	8	9	Y
A04	4	0	10	10	N
A05	1.75	1	5	2	N
A06	1	4	10	10	N
A07	2.33	4	2.67	4	N
A08	3	0	5	2	N
A09	0	1	1.33	0	N

Table S5. Phantom hand measurements for acquired amputees. Magnitude on a scale of 1-10. Y=Yes, N=No.

Supplementary discussion

P[M]HS representation in acquired amputees

In the current manuscript, while focused on congenital one-handers we also used an acquired amputee group in comparison to one-handers. In this section, we discuss the results of the P[M]HS representation analysis in acquired amputees and highlight technical considerations that should be taken under account when interpreting these results.

We previously reported local preservation of hand representation in acquired amputees' primary sensorimotor missing hand cortex (Kikkert et al., 2016; Makin, Scholz, et al., 2013), in association with phantom sensations. However, these findings were restricted to the missing hand representation in the sensorimotor cortex. We also reported large-scale changes in network-level organisation both within and outside the sensorimotor network (Makin, Filippini, et al., 2015). As such, the persistence of PHS representation following amputation is not strongly predicted. Indeed, Schmalzl, Kalckert, Ragnö, and Ehrsson, 2014 present evidence that suggests otherwise. They show that activity is observed in PHS network in response to visual stimulation, only when an illusory visual information is provided for the missing hand. Meaning that without the illusion, PHS network is not activated. This suggests that the P[M]HS representation in acquired amputees might still be anchored to the missing hand but requires the full visual illusion and experience of a hand to be activated. Alternatively, as discussed in the main text, P[M]HS representation in acquired amputees might be anchored to the effector used for interactions with the environment (handless-arm). Therefore, since amputees tend to rely on their intact hand more than their handless-arm for compensatory purposes, they show decreased P[M]HS representation compared to one-handers (who use their handless arm relatively more).

In acquired amputees, no differential P[M]HS activity was found in the PHS network areas (Figure S1). While it could be interpreted as diminished P[M]HS we advise caution when interpreting this null result. Acquired amputees comprise of a heterogeneous group, with regards to phantom sensations phenomenology, e.g. telescoping, phantom pain and phantom sensations (Table S5). Since these variables might impact P[M]HS representation, this could result in reduced power when averaging across amputees. As such, it is difficult to determine based on the current findings whether P[M]HS exists in amputees. We note that one-handers did not show greater PHS activity for their intact hand compared to one-handers, suggesting that the amputees group was not generally underpowered and as such the P[M]HS comparison presented in the main text is still valid.

References

Andersson, J. L. R., Jenkinson, M., & Smith, S. (2007a). *Non-linear optimisation FMRIB Technial Report TR07JA1*.

Andersson, J. L. R., Jenkinson, M., & Smith, S. (2007b). *Non-linear registration aka Spatial normalisation FMRIB Technial Report TR07JA2*.

Beckmann, C. F., Jenkinson, M., & Smith, S. M. (2003). General multilevel linear modeling for group analysis in FMRI. *NeuroImage*, *20*(2), 1052–1063. https://doi.org/10.1016/S1053-8119(03)00435-X

Cooke, D. F., & Graziano, M. S. A. (2003). Defensive Movements Evoked by Air Puff in Monkeys. *Journal of Neurophysiology*, *90*(5), 3317–3329.

Greve, D. N., & Fischl, B. (2009). Accurate and robust brain image alignment using boundary-based registration. *NeuroImage*, *48*(1), 63–72. https://doi.org/10.1016/j.neuroimage.2009.06.060

Hahamy, A., Macdonald, S. N., van den Heiligenberg, F., Kieliba, P., Emir, U., Malach, R., … Makin, T. R. (2017). Representation of multiple body parts in the missing-hand territory of congenital one-handers. *In Revisions*.

Hahamy, A., Sotiropoulos, S. N., Henderson Slater, D., Malach, R., Johansen-Berg, H., & Makin, T. R. (2015). Normalisation of brain connectivity through compensatory behaviour, despite congenital hand absence. *eLife*, *4*. https://doi.org/10.7554/eLife.04605

Jenkinson, M., Bannister, P., Brady, M., & Smith, S. (2002). Improved Optimization for the Robust and Accurate Linear Registration and Motion Correction of Brain Images. *NeuroImage*, *17*(2), 825–841. https://doi.org/10.1006/nimg.2002.1132

Jenkinson, M., & Smith, S. (2001). A global optimisation method for robust affine registration of brain images. *Medical Image Analysis*, *5*(2), 143–156. https://doi.org/10.1016/S1361-8415(01)00036-6

Kikkert, S., Kolasinski, J., Jbabdi, S., Tracey, I., Beckmann, C. F., Johansen-Berg, H., & Makin, T. R. (2016). Revealing the neural fingerprints of a missing hand. *eLife*, *5*. https://doi.org/10.7554/eLife.15292

Makin, T. R., Cramer, A. O., Scholz, J., Hahamy, A., Henderson Slater, D., Tracey, I., & Johansen-Berg, H. (2013). Deprivation-related and use-dependent plasticity go hand in hand. *eLife*, *2013*, 1–15. https://doi.org/10.7554/eLife.01273.01273

Makin, T. R., Filippini, N., Duff, E. P., Slater, D. H., Tracey, I., & Johansen-Berg, H. (2015). Network-level reorganisation of functional connectivity following arm amputation. *NeuroImage*, *114*, 217–225. https://doi.org/10.1016/j.neuroimage.2015.02.067

Makin, T. R., Holmes, N. P., & Zohary, E. (2007). Is That Near My Hand? Multisensory Representation of Peripersonal Space in Human Intraparietal Sulcus. *Journal of Neuroscience*, *27*(4), 731–740. https://doi.org/10.1523/JNEUROSCI.3653-06.2007

Makin, T. R., Scholz, J., Filippini, N., Slater, D. H., Tracey, I., Johansen-berg, H., & Henderson Slater, D. (2013). Phantom pain is associated with preserved structure and function in the former hand area. *Nature Communications*, *4*, 1570. https://doi.org/10.1038/ncomms2571

Makin, T. R., Scholz, J., Henderson Slater, D., Johansen-Berg, H., & Tracey, I. (2015). Reassessing cortical reorganization in the primary sensorimotor cortex following arm amputation. *Brain : A Journal of Neurology*, *138*(Pt 8), 2140–6. https://doi.org/10.1093/brain/awv161

Marcus, D. S., Harwell, J., Olsen, T., Hodge, M., Glasser, M. F., Prior, F., … Van Essen, D. C. (2011). Informatics and Data Mining Tools and Strategies for the Human Connectome Project. *Frontiers in Neuroinformatics*, *5*, 4. https://doi.org/10.3389/fninf.2011.00004

Schmalzl, L., Kalckert, A., Ragnö, C., & Ehrsson, H. H. (2014). Neural correlates of the rubber hand illusion in amputees: A report of two cases. *Neurocase*, *20*(4), 407–420. https://doi.org/10.1080/13554794.2013.791861

Smith, S. M. (2002). Fast robust automated brain extraction. *Human Brain Mapping*, *17*(3), 143–155. https://doi.org/10.1002/hbm.10062

Uswatte, G., Taub, E., Morris, D., Light, K., & Thompson, P. A. (2006). The Motor Activity Log-28: assessing daily use of the hemiparetic arm after stroke. *Neurology*, *67*(7), 1189–94. https://doi.org/10.1212/01.wnl.0000238164.90657.c2

Woolrich, M. (2008). Robust group analysis using outlier inference. *NeuroImage*, *41*(2), 286–301. https://doi.org/10.1016/j.neuroimage.2008.02.042

Woolrich, M. W., Behrens, T. E. J., Beckmann, C. F., Jenkinson, M., & Smith, S. M. (2004). Multilevel linear modelling for FMRI group analysis using Bayesian inference. *NeuroImage*, *21*(4), 1732–1747. https://doi.org/10.1016/j.neuroimage.2003.12.023

Woolrich, M. W., Ripley, B. D., Brady, M., & Smith, S. M. (2001). Temporal autocorrelation in univariate linear modeling of FMRI data. *NeuroImage*, *14*(6), 1370–86. https://doi.org/10.1006/nimg.2001.0931

Worsley, K. J. (2001). Statistical analysis of activation images. In P. Jezzard, P. M. Matthews, & S. M. Smith (Eds.), *Functional MRI: An Introduction to Methods*. OUP.
